# Oocyte zona pellucida dysmorphology is associated with diminished in-vitro fertilization success

**DOI:** 10.1186/s13048-014-0111-5

**Published:** 2015-02-27

**Authors:** May-Tal Sauerbrun-Cutler, Mario Vega, Andrzej Breborowicz, Eric Gonzales, Daniel Stein, Mathew Lederman, Martin Keltz

**Affiliations:** Continuum Reproductive Center, Department OBGYN, Mount Sinai St. Luke’s-Roosevelt Hospitals, 425 W 59th Street Suite 5A, New York, NY USA 10019

**Keywords:** In-vitro fertilization, Zona Pellucida, Infertility, Oocyte

## Abstract

**Background:**

Although irregularities in human zona pellucida (ZP) morphology are well described, there is scant literature on the clinical significance of ZP dysmorphology. We, therefore, designed a retrospective cohort trial of ZP dysmorphology to assess the clinical significance of ZP dysmorphology and its affect on IVF outcome. Over the same time period a random sample of 77 cycles of 77 subjects with all normal oocyte morphology were selected as controls.

**Methods:**

Between July 2006 and December 2010, all fresh non-donor cases performed at a university hospital IVF center were assessed for ZP dysmorphology. ZP dysmorphology included extracytoplasmic abnormalities (dark ZP and large perivitelline space) and oocyte shape (oval or irregularly shaped ZP). 136 IVF cycles from 119 subjects were identified where a majority of oocytes displayed ZP dysmorphology. Over the same time period a random sample of 77 cycles of 77 subjects with all normal oocyte morphology were selected as controls. IVF prognostic and outcome parameters were compared between the patients with dysmorphic and normal oocytes.

**Results:**

136/1710 (8.0%) cycles of fresh non-donor IVF displayed predominant ZP dysmorphology. Dysmorphic and normal oocytes showed no difference in the oocyte quality predictors such as FSH (6.03+/−2.5 vs. 6.8+/−2.3 IU/L), or AMH (2.5+/−2.0 vs. 2.30+/−1.5 ng/ml levels). ZP dysmorphology was associated with markedly diminished clinical pregnancy rates (44% vs. 70%; RR:0.62 [0.48, 0.80]; p = 0.0002), implantation rates (.17 vs. .36; IRR: 0.48 [0.34, 0.68]; p < 0.0001) and live birth rates as compared to non dysmorphic oocytes (29% vs. 52%; RR:0.55 [0.39, 0.79]; p = 0.001).

**Conclusions:**

ZP dysmorphology is associated with markedly diminished pregnancy and implantation rates in IVF. The poorer outcome appears to be independent of the usual markers of ovarian reserve.

## Introduction

Irregularities in human zona pellucida (ZP) morphology have been described in the literature. ZP Dysmorphology has an incidence of 2-5% of all oocytes [1,2]. The ZP has an important role in oocyte fertilization and when thickening is present it may prevent implantation. It is essential for sperm binding and preventing polyspermy. It may have a protective role prior to hatching and protecting embryos from mechanical stress prior to implantation.

The Atlas of Human Gametes lists ZP darkening, focal thickening, bilayering, irregular shape, as well as debris within the zona or periviteline space as ZP dysmorphology that may benefit from ICSI. Yet, there's scant literature on the clinical significance of ZP dysmorphology. IVF laboratory morphological assessment of retrieved oocytes is not used to select for fertilization nor has it been shown to predict IVF outcome.

All available oocytes that meet the basic criteria of mature (MII) undergo standard fertilization by IVF or ICSI. Embryo characteristics for transfer are based on morphological characteristics after fertilization which vary at different institutions.

De Sutter et al. and Ten et al. noted that when intracytoplasmic sperm injection (ICSI) is utilized there was no decrease in fertilization rates or embryo quality with ZP abnormalities [3,4]. In addition, Balaban et al. and had similar findings including no effect on pregnancy or implantation rates [2]. Balaban et. al used the following criteria to define oocyte dysmorphology: 1. Extracytoplasmic abnormalities (dark zona pellucida and large perivitelline space), 2. Cytoplasmic abnormalities (dark cytoplasm, granular cytoplasm, and refractile body), 3. Shape abnormalities, and 4. Multiple abnormalities (double and triple abnormalities) [2]. However, Chamayou et al. found a correlation between increased perivitelline space, presence of granulation and subsequent embryo quality, but still had no effect on clinical pregnancy and implantation rate [5]. While most studies have not documented a negative association of ZP dysmorphology with decreased IVF outcomes, we noted a predominance of dysmorphic ZP in the oocytes of several patients who had poor IVF outcome [6]. We, therefore, designed a retrospective cohort trial of ZP dysmorphology to assess its affect on IVF outcome.

## Material and methods

**Figure 1:**
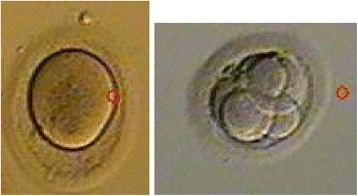
**Oval shaped ZP oocyte and embryo.**

**Figure 2:**
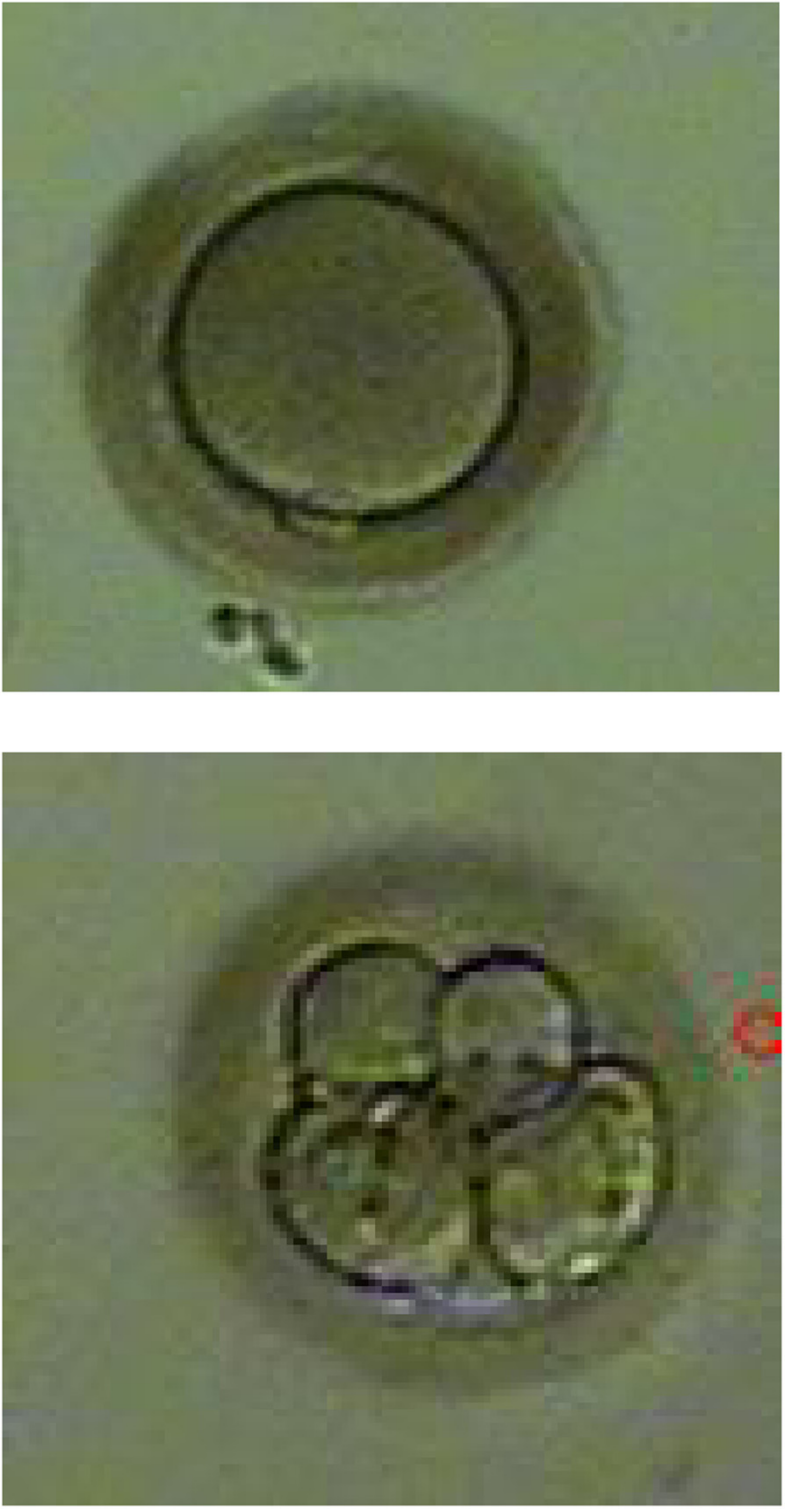
**Dark ZP oocyte and corresponding Day 3 Embryo.**

**Figure 3:**
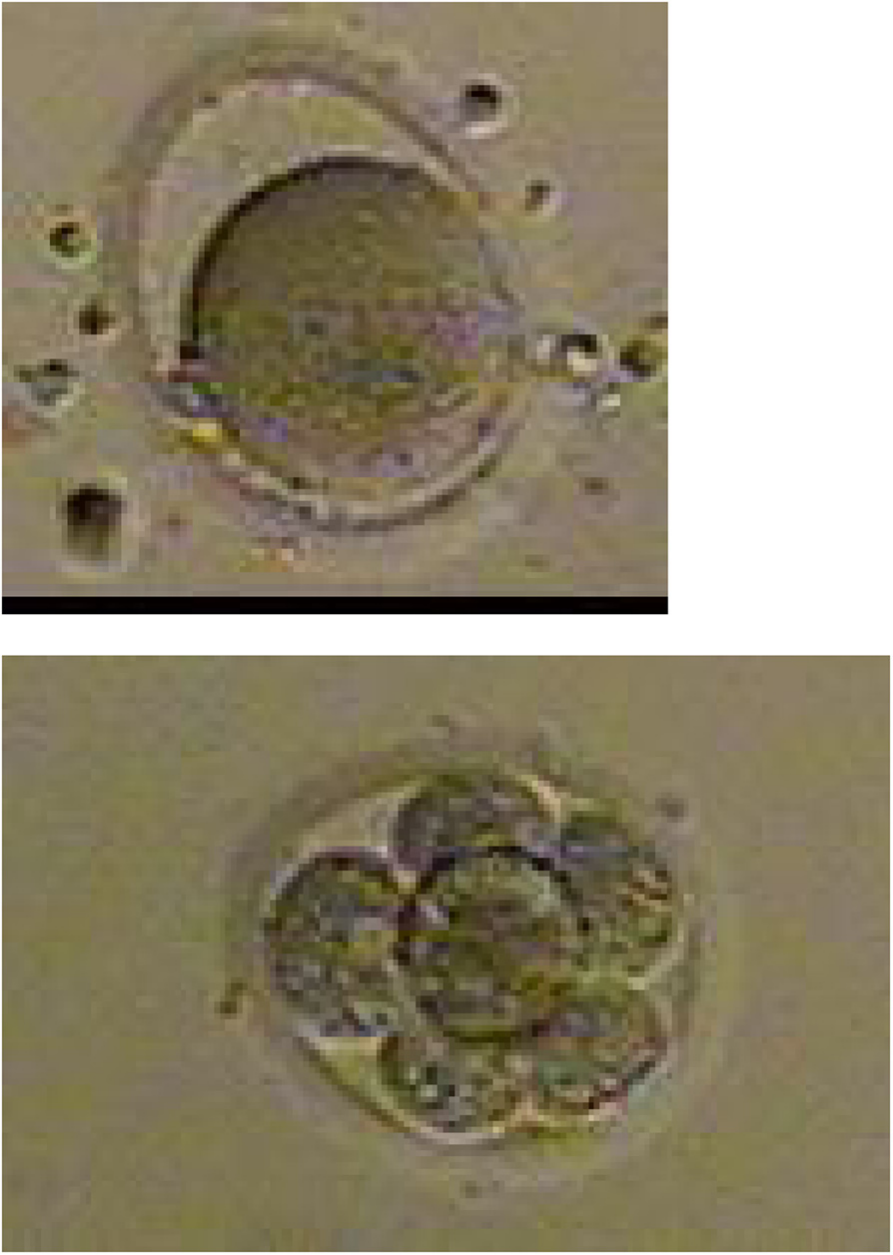
**Enlarged Perivitelline space oocyte and corresponding day 3 embryo.**

Between July 2006 and December 2010, all fresh non-donor cases preformed at a university hospital IVF center were assessed for extracytoplasmic abnormalities oocyte shape: oval or irregularly shaped ZP (Figure 1), dark ZP (Figure 2) and large perivitelline space (Figure 3) as defined by Balaban et al. [2]. IRB approval was obtained for a retrospective study.

136 IVF cycles from 119 subjects under 42 years old were identified where a majority (over 50% of oocytes) per cycle displayed one or more extracytoplasmic ZP abnormality (Figures 1, 2 and 3). Over the same time a matched control group for age of 77 cycles of 77 subjects in which all the oocyte ZP morphology was normal were selected as controls. The controls were carefully matched for age and time period of IVF to the cases to reduce confounding. One to one matching was performed for each case of dysmorphic oocyte to a control within 1 year older or 1 year younger in age. All controls were matched to cases within a week of IVF. In addition the same experienced embryologist was evaluating both cases and controls. IVF prognostic and outcome parameters were compared between the cases and controls including age, Day 2/3 basal FSH and AMH, as well as total gonadotropin dose, peak estradiol, oocyte yield, fertilization rates, blastulation rates, oocyte development (M1 vs. M2), embryo yield, pregnancy and implantation rates, as well as live birth rates. There were 15 patients with dysmorphic oocytes that had more than one cycle. We only included their last cycle in our analysis. Oocytes were fertilized with ICSI or conventional IVF based on our standard protocol. Controlled stimulation during IVF was performed using a combination of GnRH agonists and gonadotropins, in a long or short protocol, as well as antagonist protocols for all subjects. Ovarian follicular development was monitored by transvaginal sonography and serial E2 levels. Once oocytes reached 18 mm or more in maximum diameter as viewed by sonography, 10,000 IU of hCG was administered. Thirty-five hours after hCG administration, oocytes were retrieved transvaginally under ultrasound guidance. Insemination was performed approximately 4 hours after retrieval using either 50,000 motile sperm per oocyte or intracytoplasmic sperm injection (ICSI) in the case of male factor infertility or, occasionally, oocyte factors. Prior to insemination, the oocytes were viewed under light microscopy and any abnormal morphology characteristic noted and photographs taken. Human tubal fluid (HTF) (Irvine Scientific, Irvine, CA) with 15% synthetic serum substitute (SSS) was used for embryo culture. The presence of two pronuclei (2PN) 20 hours after insemination confirmed fertilization. On day 3 after fertilization the embryos were analyzed for embryo grade, cell number, and percent fragmentation. Embryo transfer was performed day 3 vs. day 5 based on number of fertilized embryos and embryo grading. Serum beta hCG levels were drawn on days 12 and 14 after transfer. Clinical pregnancy was determined by the presence of a gestational sac by transvaginal sonography 19 or 20 days after transfer. Biochemical, nonclinical pregnancies were included in the statistical analyses as nonpregnancies. Live birth rates were based on SART registry.

### Statistical analysis

Descriptive statistics of mean and standard deviation were computed for continuous patient outcome measures and compared between patient groups with dysmorphic and normal oocytes using a student’s t-test. Blastulation, fertilization, implantation and live birth rates were computed and corresponding Incidence Rate Ratios (ratios of rates for dysmorphic to normal) were estimated and tested for difference from one using Poisson regression models. Clinical pregnancy, live birth, and miscarriage probabilities/proportions were computed and corresponding Relative Risks (ratios of proportions for dysmorphic to normal) were estimated and tested for difference from one using log binomial models. Multivariable Poisson and log binomial regression models were used to estimate age-adjusted relative risks and incidence rate ratios and ANCOVA was used to estimate age-adjusted differences in the average number of embryos transferred and fertilized. Agonist, Antagonist and Flare cycle distributions were compared between dysmorphic and normal oocyte patient groups using a two sample Chi-Square test. All statistical analyses were performed using Systat Version 13 and SAS Version 9.2 (SAS Institute, Inc., Cary, NC). All hypothesis testing was conducted at the 5% level of significance.

## Results

136/1710 (8.0%) cycles of fresh non-donor IVF displayed predominant ZP dysmorphology. Patients with Dysmorphic ZP and normal oocytes were 1.2 years older on average, however showed no difference in the oocyte quality predictors such as FSH and AMH levels or in percentage of patients with a poor response defined as less than 5 oocytes obtained at retrieval. There was still no difference in oocyte quality predictors even when we adjusted for age (Table 1). In both Tables 1 and 2 we present age adjusted estimates and because there are no difference in most of our outcomes we're reporting the following results as unadjusted estimates. Patients with dysmorphic ZP and normal oocytes also showed no significant difference in their mean (SD) percent of mature (M2) oocytes retrieved (78.0% (17.9%) vs. 81.3% (17.9%)). Patients with dysmorphic ZP required significantly more gonadotropins, had borderline significant lower peak estradiol levels, fewer long protocol cycles and on average produced 2.5 fewer oocytes per cycle (10.3 (5.4) vs. 12.8 (5.4); p = 0.002).

**Table 1:** **Oocyte quality predictors**

	**Dysmorphic oocytes**	**Normal oocytes**	**Dysmorphic vs. Normal**	**Dysmorphic vs. Normal oocytes** ^**4**^
			**Diff** ^**1**^ **/RR** ^**2**^ **/ IRR** ^**3**^ **[95% CI]**	**Difference** ^**1**^ **/RR** ^**2**^ **/ IRR** ^**3**^ **[95% CI]**
	**N = 119**	**N = 77**	**P-value**	**P-value**
**Age**	35.5 (3.9)	34.3 (4.1)	1.2 [0.05,2.4]	
Mean (SD)			P = 0.040	
**AMH** ng/ml	2.5 (2.0)	2.3 (1.5)	0.2 [−0.9, 1.2]	−0.1 [−1.2, 0.9]
Mean (SD)			P = 0.762	P = 0.795
**FSH** IU/l	6.0 (2.5)	6.8 (2.3)	−0.8 [−1.7, 0.2]	−0.8 [−1.7, 0.2]
Mean (SD)			P = 0.115	P = 0.120
**Total Gonadotropin dose** IU	3847.4 (1791.8)	3128.2 (1842.4)	719 [184, 1254]	525 [12, 1038]
Mean (SD)			P = 0.009	P = 0.045
**Peak Estradiol** Pg/ml	2760.6 (1346.4)	3157.4 (1377.6)	−396.8 [−802.7, 9.1]	−345.5 [−753.8, 62.8]
Mean (SD)			P = 0.055	P = 0.097
**Oocytes retrieved per cycle**	10.3 (5.4)	12.8 (5.4)	−2.5 [−4.0, −0.9]	−2.2 [−3.7, −0.6]
Mean (SD)			P = 0.002	P = 0.007
**% M2 oocytes**	78.0% (17.9%)	81.3 (17.9%)	−3.3% [−8.4%, 1.9%]	−3.4% [−8.6%, 1.8%]
Mean (SD)			P = 0.214	p = 0.196
**Long Agonist cycles**	45%	68%	0.005	
**Short Antagonist cycles**	25%	18%		
**Short Flare cycles**	31%	14%		

^1^Difference estimates used to compare means (SD).

^2^Relative Risk (RR) estimates used to compare probabilities.

^3^Incidence Rate Ratio (IRR) estimates used to compare rates.

^4^Age Adjusted estimates.

**Table 2:** **IVF outcomes**

	**Dysmorphic oocytes**	**Normal oocytes**	**Dysmorphic vs. Normal oocytes**	**Dysmorphic vs. Normal oocytes** ^**4**^
	**N = 119**	**N = 77**	**Difference** ^**1**^ **/RR** ^**2**^ **/ IRR** ^**3**^ **[95% CI]**	**Difference** ^**1**^ **/RR** ^**2**^ **/ IRR** ^**3**^ **[95% CI]**
			**P-value**	**P-value**
**Transferred embryo per cycle**	3.1 (1.2)	2.7 (1.2)	0.4 [0.06, 0.8]	0.25 [−0.07, 0.57]
Mean (SD)			P = 0.021	P = 0.128
**Embryos fertilized per cycle**	6.35 (3.68)	8.47 (3.98)	−2.1 [−3.2, −1.0]	−1.91 [−3.00, −0.81]
Mean (SD)			P < 0.001	P < 0.001
**Blastulation rate per cycle**	.26	.42	IRR = 0.61 [0.51, 0.73]	IRR = 0.68 [0.56, 0.82]
**blasts/embryos fertilized**	202/756	274/652	P < 0.001	P < 0.001
**Fertilization rate per cycle**	.85	.85	IRR = 0.99 [0.90, 1.11]	IRR = 1.00 [0.90, 1.11]
**fertilized /oocytes inseminated**	832/ 891	652/766	P = 0.953	P = 0.935
**Implantation rate**	.17	.36	IRR = 0.48 [0.34, 0.68]	IRR = 0.52 [0.37, 0.73]
**sacs/embryos transferred**	61/354	72/202	P < 0.001	P < 0.001
**Clinical Pregnancy rate per cycle**	44%	70%	RR = 0.62 [0.48, 0.80]	RR = 0.64 [0.49, 0.82]
	52/119	54/77	P < 0.001	P < 0.001
**Live Birth rate per cycle**	29%	52%	RR = 0.55 [0.39, 0.79]	RR = 0.60 [0.42, 0.87]
	34/119	40/77	P = 0.001	P = 0.007
**Miscarriage rate**	35%	26%	RR = 1.34 [0.74, 2.40]	RR = 1.28 [0.73, 2.25]
	18/52	14/54	P = 0.333	P = 0.396

^1^Difference estimates used to compare means (SD).

^2^Relative Risk (RR) estimates used to compare probabilities.

^3^Incidence Rate Ratio (IRR) estimates used to compare rates.

^4^Age Adjusted estimates.

N: number of patients in each group equivalent to number of cycles.

Patients with dysmorphic ZP produced on average 2.1 fewer embryos (6.4 (3.7) vs. 8.5 (4.0); p < 0.001) with similar rates of extended embryo culture compared to the normal oocyte group. Patients with dsymorphic ZP also had lower blastulation with equivalent fertilization rates and on average 0.4 more transferred embryos per cycle (3.1 (1.2) vs. 2.7 (1.2); p = 0.021). All of our ZP abnormalities persisted from oocyte to embryo except for the enlargement of the perivitelline space. Forty-four percent of patients with ZP dysmorphology had a clinical pregnancy while 70% of patients with normal oocytes had a clinical pregnancy. This represents a 38% reduction in the likelihood of clinical pregnancy in patients with ZP dysmorphology (RR:0.62 [0.48, 0.80]; p < 0.001). The implantation rate of 0.17 in dysmorphic patients was significantly lower than the implantation rate of 0.36 in normal patients. This represents a 52% reduction in the implantation rate of dysmorphic patients (IRR: 0.48 [0.34, 0.68]; p < 0.001). Similarly, 29% of dysmorphic patients experienced a live birth while 52% of normal oocyte patients had a live birth. This represents a 45% reduction in the likelihood of a live birth in dysmorphic patients (RR:0.55 [0.39, 0.79]; p = 0.001). Results are displayed in Tables 1 and 2.

## Discussion

ZP dysmorphology is associated with slightly fewer oocytes and embryos as well as markedly diminished pregnancy and implantation rates in IVF. Live birth rates were also almost half the amount of normal oocytes. The poorer outcome appears to be independent of the usual markers of ovarian reserve, however the dysmorphic oocytes were more likely to require more gonadotropins in short agonist and antagonist cycles indicating these patients showed a poorer response to stimulation.

It is unclear why ZP dysmorphology may impair oocyte function however one possible theory is that ZP dymorphology is a marker for poorer oocyte quality related to endometriosis. Studies have shown endometriosis patients to have poorer oocyte quality because of a higher apoptotic incidence, more alterations of the cell cycle, and a higher incidence of oxidative stress than patients with any of the other infertility causes (tube, male, and idiopathic factors) [7,8]. We initially became interested in oocyte ZP dysmorphology as several patients with severe endometriosis had misshapen zona pellucida. An initial published abstract suggested an association between ZP dysmorphology and endometriosis, but further work would be required to confirm any causal link. Another interesting avenue for research would be to link cytokines associated with endometriosis with oocyte ZP dysmorphology.

Another theory is that alterations in ZP morphology may be caused by patterning problems of the glycoprotein matrix encoded by ZP 1, 2 and 3 genes. Sterility in mice is associated with mutations in ZP genes and fertilization problems have been found in patients with ZP1 and ZP3 gene variations [9-12]. In human oocytes two sequence variations of ZP3 genes were more frequent in oocytes with abnormal zona in comparison to those with normal zona [12]. Therefore these morphologic mutations may be linked to polymorphisms in the ZP gene although more research needs to be performed to confirm this association.

In addition the spherical shape of the ZP ensures maximal contact between blastomeres of embryos. Therefore, cleaving embryos from ovoid oocytes may have a reduced chance to express an optimal cell association. Ebner et al. [13] described ovoid zona favoring generation of atypical cleavage patterns resulting in delayed compaction and blastocyst formation. This finding may explain why the dysmorphic oocytes had decreased blastulation rates in comparison to our control group

Our results are different than those published by Balaban and Sutter where no differences were noted in IVF outcomes. This difference may be explained by the criteria used to evaluate oocyte morphology in the present study. While Balaban looked at extracytoplasmic and cytoplasmic abnormalities, our study primarily looked at extracytoplasmic ZP abnormalities and oocyte shape. Therefore, the differences in our finding may be attributed to our emphasis on ZP in comparison to cytoplasmic abnormalities used by Balaban et al. Rienzi et al. also found that large perivitelline space correlated with lower reproductive outcomes and developed an oocyte morphology scoring system incorporating large perivitelline space along with other intracytoplasmic abnormalities into their scoring system. They found an inverse relationship between their oocyte morphological scoring system and pregnancy rates [1,14]. Similar to our study there was a finding of poorer IVF outcome.

Our findings support that selecting oocytes with no dysmorphic characteristics may yield improved outcomes as is currently common practice in many IVF centers. Furthermore, cycles with dysmorphic oocytes may be considered less favorable. Oocyte morphology assessment may be a useful adjunct to later embryo assessment and may aid in our decision making about how many embryos to transfer. Most of our abnormal oocytes and controls were inseminated with ICSI and assisted hatching as over 90% of cycles at this time were being performed by ICSI in our lab. There was no significant difference in percentage of assisted hatching or ICSI between the ZP dysmorphic and the normal group. Future studies are necessary to evaluate if ICSI affects clinical outcomes for abnormal oocytes in comparison to standard IVF [15]. As ICSI is more commonly performed in our lab, we were are unable to answer this question from this study.

One limitation of our study is that as a retrospective study we cannot confirm a causal link between the worse IVF outcomes and ZP dysmorphology. However, we reduced confounding variables by using a control group of cycles matched in timing and utilizing the same embryologist for both the dysmorhpic and normal oocytes. When we matched our cases to controls we tried to reduce bias by matching oocytes with ZP abnormalities to normal oocytes by IVF cycles within one week of each other and by patient age within 2 years (1 year older or 1 year younger). Our results showed age matched controls that were statistically younger therefore it is possible that the lower ages in the control group with normal ZP features may have contributed to their favorable IVF outcomes in comparison to the dysmorphic group. However when we statistically corrected for age there was no difference in our oocyte quality predictors or implantation and clinical pregnancy rates. 15 patients in the dysmorphic oocyte group had more than one cycle and we only included their last cycle in our analysis in order to reduce bias because data from the same patient, although from different cycles are technically not independent. However, when we included them in our calculation initially the outcomes were all the same. In future studies we will look for possible causes of ZP dysmorphology such as endometriosis, pelvic infection and chronic pelvic pain.
